# Demographics of Lower Limb Amputations in the Pakistan Military: A Single Center, Three-Year Prospective Survey

**DOI:** 10.7759/cureus.566

**Published:** 2016-04-11

**Authors:** Farooq A Rathore, Saeed B Ayaz, Sahibzada N Mansoor, Ali R Qureshi, Muhammad Fahim

**Affiliations:** 1 Department of Rehabilitation Medicine, CMH Lahore Medical College and Institute of Dentistry; 2 Department of Rehabilitation Medicine, Combined Military Hospital, Okara, Pakistan; 3 Department of Rehabilitation Medicine, Combined Military Hospital, Panoaqil, Pakistan; 4 Department of Rehabilitation Medicine, Combined Military Hospital, Karachi, Pakistan; 5 Department of Rehabilitation Medicine, KRL Hospital, Islamabad

**Keywords:** amputation, limb amputation, epidemiology, demography, pakistan, bombblast, combat injury, injuries, injury

## Abstract

**Introduction:**

The Pakistan military has been actively engaged in the war against terror for more than a decade. Many officers and soldiers have lost their limbs in this war. But the data on traumatic lower limb amputations in Pakistan is sparse. The aim of this study is to prospectively document the epidemiological profile of lower limb military amputees presenting at the largest rehabilitation centre of Pakistan over a three-year period.

**Materials & methods:**

A prospective three-year survey was conducted at the Armed Forces Institute of Rehabilitation Medicine (AFIRM), Pakistan. One hundred twenty-three consecutive patients with lower limb amputations were enrolled in the survey. The demographic data, etiology, associated injuries, complications profile, and type of prosthesis provided were documented. The data analysis was done using the statistical analysis tool SPSS V 20 (IBM®,NY, USA).

**Results:**

All patients were male. Most had traumatic amputation (119), were between 20–40 years (106), with unilateral amputation (115). Mine blast injury was the leading cause in 73 (59.3%) and most (58.5%) were fitted with modular prosthesis. Transtibial amputation was the commonest level (65), followed by transfemoral (30). The time of surgical amputation was not documented in 87% of the patients. Half of the patients (54%) had associated injuries. Seventy-nine patients had at least one complication with phantom pain being the commonest in 25% cases.

**Conclusions:**

This is the largest prospective demographic survey of lower limb amputees in Pakistan military to date. Scores of soldiers and civilians in Pakistan have suffered lower limb amputation. The availability of demographic data can improve the trauma and rehabilitation services for better understanding and management of such cases. There is a need to conduct large scale community-based epidemiological surveys to direct future policies and develop amputee rehabilitation services in the public sector.

## Introduction

Amputation is one of the oldest surgical procedures known to humankind. Historically, it was done with no regard to pain control or aseptic measures. Because of the advances in surgery, anesthesia, and rehabilitation medicine in the last century, amputees now have better pre- and postoperative care, access to multidisciplinary rehabilitation and provision of excellent quality prosthetics, resulting in good functional outcome and community reintegration.

Different aspects of amputee management, such as epidemiology, complications profile, quality of life (QOL) and functional outcomes, have mainly been reported from developed regions of the world, with a paucity of data from low resourced countries. The burden of amputees in Pakistan is on the rise due to the different acts of terrorism and suicide bombings during the last decade. The armed forces, being in the front line of the war on terror, have suffered the most and lost thousands of soldiers, whilst hundreds more have sustained long-term permanent disabilities including amputation. This study is an attempt to prospectively document the epidemiological profile of lower limb amputees presenting at the largest rehabilitation centre of Pakistan over a three-year period. The aim is to identify the demographics of traumatic lower limb amputation in order to formulate better management and rehabilitation plans and guide the policy makers in addressing long-term rehabilitation and community reintegration of amputees.

## Materials and methods

Permission from hospital administration was obtained and data collection commenced on August 1, 2007. Consecutive male and female patients, over 18 years of age, with any etiology, reporting to the outpatient department of Armed Forces Institute of Rehabilitation Medicine (AFIRM) with minor and major amputations of lower limbs were enrolled. The study was conducted in a military institution where all patients were army soldiers. As part of the admission process, every patient signed a document granting the hospital the right to treat them, collect data, and include them in teaching activities of medical students and residents. Therefore only verbal informed consent was obtained after the importance of the study and its possible positive impact on the community was explained to them. All patients gave consent to participate. Demographic data (age, gender, educational status, marital status, and etiology) were recorded and patients were examined for site and level of amputation, presence of complications, and type of prosthesis provided. Since the patients reported from different hospitals and were unable to recall all events leading to amputation, we had to rely on the medical documents and records available with the patient. The available documents were scanned for documentation of complications (specifically phantom limb sensation and pain) and timing of surgery.

Data was entered manually in data forms and entered in SPSS V20 for statistical analysis. Frequencies and means were calculated for quantitative data.

## Results

Our survey included 123 male patients, over the age of 18, who reported with minor or major lower limb amputation over the three-year period (August 1, 2007–August 31, 2010). All were military personnel. Almost all (119, 96.7%) had a traumatic etiology for their amputation, mine blast injury being the most common (59.3%). Other causes included injury by multiple shrapnels, direct hit by a rocket/ missile, crush injury, fall from a moving train ( when attempting to board it), and limb being crushed by a moving tank. The majority were married, aged between 21–30 years, matriculated (62.6%), and had a transtibial amputation (52.8%). Detailed demographics are presented in Table 1.

**Table 1:** Demographic Profile of Patients with Lower Limb Amputations in This Study (n= 123) RTA - road traffic accident, GSW - gun shot wound.

Gender	
Males	123 (100%)
Females	0 (0%)
Age groups (years)	
11-20	10 (8.1%)
21-30	71 (57.7%)
31-40	35 (28.5%)
41-50	07 (5.7%)
Educational status	
Under matric	30 (24.4 %)
Matriculate	77 (62.6%)
High school	10 (8.1%)
Graduates	06 (4.9%)
Cause of amputation	
Mine blast injury	73 (59.7%)
Other traumatic causes	23 (18.7%)
RTA	13 (10.6 %)
GSW	11 (8.9 %)
Non traumatic	4 (3.3%)
Level of amputations	
Transfemoral	30 (24.4 %)
Through knee/knee disarticluation	4 (3.3%)
Transtibial	65 (52.8%)
Symes	9 (7.3%)
Chopart's	5 (4.1%)
Lisfranc injury	3 (2.4 %)
Toe	3 (2.4%)
Other	4 (3.3%)
Side of amputation	
Right	55 (44.7%))
Left	60 (48.8%)
Bilateral	08 (6.5%)
Time of surgical amputation	
Within 12 hours	4
Within 24 hours	9
1-5 days	1
After 5 days	1
Not documented	108
Type of prosthesis provided	
Conventional	40 (32.5 %)
Modular	72 (58.5 %)
Shoe inserts & modifications	11 (8.9%)

The majority of amputees enrolled in this study had sustained injuries during military operations and were evacuated to different settings. The authors could only rely on available documents to ascertain timing of surgery in these patients. This information was not documented in 87.8% of the cases, highlighting an oversight of the medical documentation system in war situations. More than half (53.7%) had associated problems including soft tissue loss, long bone fracture, abdominal injury, loss of vision or hearing. Of the surveyed patients 79 reported at least one complication. Most frequent complications were phantom limb sensation, neuroma formation, wound dehiscence, and contracture formation (Table 1). Seventy-two patients were provided with a modular prosthesis, while shoe inserts and modifications were provided in 11 cases. The other patients were fitted with a conventional prosthesis.

## Discussion

Loss of a limb not only affects mobility, self-care activities and earning capability but also human psychology, social relations, and avocational interests. There are many etiologies for limb loss and statistics may vary in different countries, populations or regions [1]. In Pakistan, a difference between military and civilian populations in amputation epidemiology has been reported. In a study carried out in Karachi, Humail et al. reported vascular disease to be the major cause of amputation (63%) in the civilian population, followed by trauma (23.28%) and tumors (13.69%) [2]. A study on military population by Razzaq et al., reported that 99% of amputations were caused by trauma and 1% due to tumors [3]. In this study, 96.7% (n=119) of subjects had a traumatic etiology. The dissimilarity may be attributed to the recent surge in acts of terrorism in the last decade. The increased incidence of suicide bombings, blasts due to land mines, improvised explosive devices and bullet injuries have resulted in an increased number of traumatic amputations in military personnel during military operations and terrorist attacks.

Globally a different pattern of amputations has been observed between the developed and developing nations [4]. In the developed countries, vascular diseases, diabetes mellitus and tumors account for 68% of all amputations performed each year [5]. In the developing world, trauma is the leading cause of amputation that can account for up to 80% of all amputations, particularly in countries with a recent history of warfare or civil unrest [4].

Most of the amputees (57.7%) in this study were 21–30 years old. A previous study at the same centre reported a mean age of 30±9 years [3]. Traumatic amputees in a study from Chile had a mean age of 35.5 years (range 17–63 years) [6]. In Denmark, Ebskov, et al. found a mean age of 44.8 years amongst males with traumatic lower limb amputations [7]. Pezzin, et al. reported a mean age of 32 years in the USA [8]. This highlights the importance of a comprehensive multidisciplinary amputee rehabilitation program and provision of adequate prosthesis, which would allow young amputees to successfully reintegrate into the community as useful members of society. Adequate and timely amputee rehabilitation also ensures a fully functional mobility level and participation in sports activities, which is essential to all amputees and more so for a young amputee in the second–third decade of life.

Land mine blast was the most frequent cause (59.3%) of amputation in this study. This is similar to the amputee database of the Indian Armed Forces at the Artificial Limb Centre (ALC), Pune, India, where 47,000 amputees were analyzed between 1944 to 2003. Amongst traumatic etiology, mine blast injuries constituted the largest number of cases (41.74%), followed by road traffic accident (21.79%), gunshot injuries (9.29%) and railway accidents (6.78%) [9]. Dogan et al., from the war-affected eastern region of Turkey, reported gunshot injuries (n=45) as the leading etiology, followed by land mine (n=33) and hand grenade (n=7) blasts in a cohort of 177 traumatic amputees [1]. According to the US military database, 1286 amputations were recorded between September 2001 and January 2009, out of which 643 (50%) were wounded by improvised explosive devices (IEDs) [10]. A study in Sri Lanka found antipersonnel mines to be the leading cause of amputations in war-related amputations from two different localities (95% and 62.4% respectively), whilst gunshot injuries were 25.8% and 11.8% respectively [11].

The majority of studies have reported transtibial and transfemoral as the most frequent level in traumatic amputation. A large study carried out at ALC (India), reported transtibial amputations in 67.78%, followed by transfemoral amputation in 18.84% respectively [9]. Rotter, et al. reported a greater prevalence of transtibial (47%) and transfemoral (40%) amputations [6]. Other studies carried out in Cambodia and Sri Lanka showed a prevalence of 63% to 73% for transtibial amputation in mine-wounded amputees [11]. In all reported studies from war zones and conflict areas, the commonest etiologies were mine blasts and IEDs, where mines and IEDs were used increasingly in guerilla warfare. In this study transtibial amputation was the most frequent level (52.8%), followed by transfemoral (24.4%), partial foot (6.5%), Symes (7.3%), and knee disarticulation in 3.3% patients. The variety of amputation at different levels is probably due to the increased use of IEDs and mines by terrorists.

More than half the sample (53.7%) in this study had associated problems such as soft tissue loss, long bone fracture, abdominal injuries, loss of vision or hearing. The most frequent complications were wound dehiscence, phantom limb sensations, painful neuromas and contracture formation. Rotter, et al. reported a similar complication profile. Early complications included wound dehiscence (9.4 %), superficial infections (14.6%), and deep infections (26%), whilst late complications included soft tissue lesions (34.1%), painful neuroma (12.5%), phantom limb pain (12.5%), and exostosis (3.6%) [6]. A study on mine blast victims in the Indian Armed Forces reported patients with wound infection (36.36%), wound dehiscence (18.18%), and discharging sinus (9.09 %) [11].

Pakistan is currently at the forefront of the war against terrorism, and as a result, has been severely affected by a rising number of patients with traumatic conditions and disabilities. Both civilians and security forces have been affected. The number of amputees in the last decade has increased. Although there are a few centres in the public and private sectors providing prosthetic services, there is generally a lack of comprehensive multidisciplinary amputee rehabilitation services within the majority of them. Moreover there is no central registry for amputees nor availability of consolidated data for future developments and problem identification. These short comings, however, can be addressed by increasing awareness amongst policy makers and the development of these services through support from government and non-governmental organisations. Implementation of an early and efficient referral system to a rehabilitation unit for prosthetic fitting is required at the national level. There is also a need to establish amputee peer support groups to facilitate community reintegration and to counter the stigma of disability.

The Armed Forces of Pakistan have taken a keen interest in the comprehensive multidisciplinary rehabilitation of amputees in order to reintegrate them into society as active members. AFIRM is offering state-of-the-art upper and lower limb prosthetics, including bionic hands and C-legs to veteran amputees, along with vocational counseling and training in skills necessary for financial independence. In 2013 the first amputee sports gala was successfully organized and included events such as football, basketball, track events, and wall climbing.

There are some limitations in the study. The study was carried out at one center on military soldiers who have a predominantly traumatic etiology. These results cannot be generalized for the whole population. Hence, further research is needed to identify the magnitude of the problem and epidemiology, and to ascertain the most frequent type of amputation and to take preventive measures accordingly. There is a need to conduct large scale multicentre or population-based epidemiological surveys to assess the true magnitude of this issue in Pakistan. Furthermore, QOL issues and functional outcome measures need to be researched in the military and civilian environments.

## Conclusions

This study is the largest, prospective, demographic survey of lower limb amputees in Pakistan. Mine blast injury is the most common cause of amputation in Pakistani military personnel, who are aged between 21 and 30 years. Transtibial amputation is the commonest level of amputation. Most of the amputees have associated injuries and a significant percentage develop complications, the most common of which were phantom limb sensation and painful neuromas. There is a need to conduct large scale community-based epidemiological surveys to direct future policies and develop amputee rehabilitation services in the public sector. Amputee rehabilitation is one of the most successful physical medical rehabilitation interventions resulting in improved functional outcome, QOL, and better community reintegration. The war on terror has resulted in a large number of amputees in Pakistan and Pakistani physiatrists are trying their best to make a difference in the lives of these disabled individuals. 

## References

[1] Doğan A, Sungur I, Bilgiç S, Uslu M, Atik B, Tan O, Ozgökçe S, Uluç D, Coban H, Türkoğlu M, Akpinar F (2008). Amputations in eastern Turkey (Van): a multicenter epidemiological study. [Article in Turkish]. Acta Orthop Traumatol Turc.

[2] Humail SM, Ilyas S, Baqai FU (2004). Diabetic foot: major cause of lower Limb amputations. J Surg Pak.

[3] Razzaq S, Mansoor SN, Rathore FA, Akhter N, Yasmeen R (2013). Functional outcomes following lower extremity amputation at the armed forces institute of rehabilitation medicine using lower extremity functional scale. Pak Armed Forces Med J.

[4] Fergason J, Keeling JJ, Bluman EM (2010). Recent advances in lower extremity amputations and prosthetics for the combat injured patient. Foot Ankle Clin.

[5] Esquenazi A, Meier RH 3rd (1996). Rehabilitation in limb deficiency. 4. Limb amputation. Arch Phys Med Rehabil.

[6] Rotter K, Sanhueza R, Robles K, Godoy M (2006). A descriptive study of traumatic lower limb amputees from the Hospital Hel Trabajador: clinical evolution from the accident until rehabilitation discharge. Prosthet Orthot Int.

[7] Ebskov LB (1994). Trauma-related major lower limb amputations: an epidemiologic study. J Trauma.

[8] Pezzin LE, Dillingham TR, MacKenzie EJ (2000). Rehabilitation and the long-term outcomes of persons with trauma-related amputations. Arch Phys Med Rehabil.

[9] Singh BG, Pithawa AK, Ravindranath G (2009). Study of disabled treated at artifical limb centre. Med J Armed Forces India.

[10] H H (2016). A guide to U.S. Military casualty statistics: Operation Freedom’s Sentinel, Operation Inherent Resolve, Operation New Dawn, Operation Iraqi Freedom, and Operation Enduring Freedom. http://www.fas.org/sgp/crs/natsec/RS22452.pdf.

[11] Harjai M, Agarwal D, Dave P, Jog S, Arora P (2005). Mine blast injuries—our experience. Med J Armed Forces India.

